# Physico-chemical properties of calcium silicate-based sealers in powder/liquid and ready-to-use forms

**DOI:** 10.1590/0103-6440202204832

**Published:** 2022-10-21

**Authors:** Ana C P Janini, Lauter E Pelepenko, Brenda P F A Gomes, Marina A Marciano

**Affiliations:** 1 Department of Restorative Dentistry, Piracicaba Dental School, University of Campinas, Piracicaba, Sao Paulo, Brazil

**Keywords:** calcium silicate materials, endodontics, physicochemical properties, root canal sealer

## Abstract

Calcium silicate-based root canal sealers have been developed in powder/liquid or premixed ready-to-use forms. The evaluation of the physico-chemical properties of a prototype powder/liquid MTApex Sealer (Ultradent) in comparison to a ready-to-use material EndoSequence BC Sealer (Brasseler) was performed. The paste/paste epoxy resin-based AH Plus (Dentsply) served as control for comparisons. The sealers were evaluated (n = 6) regarding setting time (in dry and moist environments), flow and radiopacity, following the ISO-6876/2012 standard. Also, the pH was assessed. Material’s surface and chemical characterization was evaluated using scanning-electron-microscopy (SEM) and energy-dispersive-spectrometry (EDS). Mixed ANOVA, Shapiro-Wilk, Levene, and *post-hoc* analysis with Bonferroni correction were performed at a significance level of 5%. MTApex Sealer exhibited the highest flow and EndoSequence BC Sealer had a significantly longer setting time in dry compared to the moist environment; however, for MTApex Sealer and AH Plus no significant changes occurred when additional moisture was provided. All materials exceeded 7 mm Al of radiopacity and showed a decreasing alkalinity over the 21 day-analysis. SEM/EDS evaluation resulted in peaks of calcium, silicon, and the respective radiopacifier. The prototype powder/liquid MTApex Sealer had the highest flow and similar setting time in both dry and moist environments; opposingly, EndoSequence BC Sealer was crucially influenced by external moisture. This suggests that the powder/liquid materials’ setting seems to be more predictable.

## Resumo

Os cimentos endodônticos obturadores à base de silicato de cálcio foram desenvolvidos em formas pó/líquido ou pré-misturadas prontas para o uso. Foi realizada a avaliação das propriedades físico-químicas de um protótipo pó/líquido MTApex Sealer (Ultradent) em comparação com um material pronto para uso EndoSequence BC Sealer (Brasseler). O cimento obturador pasta/pasta à base de resina epóxi AH Plus (Dentsply) serviu de controle para as comparações. Os cimentos foram avaliados (n = 6) quanto a: tempo de presa (em ambientes secos e úmidos), escoamento e radiopacidade, seguindo a norma ISO-6876/2012; como também o pH. A superfície do material e a caracterização química foram realizadas utilizando a microscopia eletrônica de varredura (MEV) e espectrometria dispersiva de energia (EDS). ANOVA, Shapiro-Wilk, Levene e análise post-hoc com correção de Bonferroni foram realizadas com nível de significância de 5%. O MTApex Sealer exibiu um escoamento mais alto e o EndoSequence BC Sealer teve um tempo de presa significativamente mais longo em ambiente seco em comparação com o ambiente úmido; entretanto, para MTApex Sealer e AH Plus, nenhuma mudança significativa ocorreu quando umidade adicional foi fornecida. Todos os materiais excederam 7 mm de Al de radiopacidade e mostraram uma alcalinidade decrescente ao longo dos 21 dias de análise. A avaliação MEV/EDS resultou em picos de cálcio, silício e respectivo radiopacificador. O protótipo de pó/líquido MTApex Sealer teve o maior escoamento e tempo de presa semelhante em ambientes secos e úmidos; ao contrário, o EndoSequence BC Sealer foi influenciado de maneira crucial pela umidade externa. Isso sugere que a configuração dos materiais em pó/líquido parece ser mais previsível.

## Introduction

The root canal system obturation is a crucial step for the long-term success of endodontic treatments as its main objective is to three-dimensionally fill the internal anatomical structures, and, consequently, prevent future re-infections by microorganisms ensuring the periapical tissue health ^1^. The physicochemical properties of root canal sealers have both an impact on the quality of the ﬁnal root ﬁlling and a biological importance ^2^.

The handling properties of these materials and their clinical behavior may be initially evaluated *in vitro* prior to their clinical use; thus, the physical properties of various endodontic sealers according to these standards have been extensively studied, including the setting time, ﬂow and radiopacity. A longer setting time allows a sealer to penetrate intricate root canal morphology more readily after its placement, whereas a faster setting material may be used in time-sensitive situations, such an immediate retention post placement. Additionally, an adequate flow potentially allows the material to fill the anatomical irregularities in the root canal system. Finally, the material radiopacity ought to be enough to allow the distinction between adjacent anatomical structures such as bone and structural dental tissues ^3^.

Hydraulic calcium silicate-based endodontic sealer is a category of material that, in addition to filling, also interacts with the surrounding tissues through properties inherent to its composition ^4^. These sealers provide an *in situ* alkaline pH, release of calcium ions and precipitate calcium hydroxide after its hydration, chemically resulting in hydroxyapatite deposition. According to the International Organization for Standardization standard, endodontic sealers must have a maximum of 3% solubility, this level was stablished to maintain the material long-term sealing properties in the clinical scenario ^5^. The currently available calcium silicate-based sealers have either powder/liquid or premixed ready-to-use forms, and the hydration reaction occurrence is crucial for these materials to exert its properties. In powder/liquid materials, this reaction is started in the presence of water which is provided during material manipulation previously to the root canal insertion ^4^. Therefore, this category of materials in a powder/liquid form seems to be more predictable in terms of setting when compared to ready-to-use materials, whose hydration uniquely depends on the moisture in the root canal or the dentin ^6^.

AH Plus (Dentsply DeTrey, Konstanz, Germany) is an epoxy resin-based endodontic sealer, available in a paste-paste form ^7^. Its thermal curing poly-addition reaction starts immediately after the two pastes are mixed, resulting in high-molecular-weight addition polymers. Several studies have evaluated its solubility properties, adhesion to dentin, sealing ability, antimicrobial properties, cytotoxicity, and long-term clinical outcome with tomographic evaluation ^3^ ^,^ ^8^. As this endodontic sealer is widely studied, further studies comparing this material to newly developed ones would be valuable.

EndoSequence BC Sealer (Brasseler, Savannah, Georgia, USA) is a premixed ready-to-use root canal sealer available since 2008; therefore, comparisons with this material also seems to be valuable for new compositions. Previous studies have evaluated its physical, chemical, and biological properties reporting adequate results in terms of biocompatibility, release of calcium ions, alkalinity, dimensional stability and radiopacity ^9^ ^,^ ^10^.

MTApex Sealer (Ultradent Products Inc., South Jordan, Utah, USA) is a newly developed prototype endodontic sealer in a powder/liquid form with a calcium silicate base. Its powder contains tricalcium silicate as main component and tantalum oxide is used as a radio-opacifier, which does not cause dentinal staining ^11^. This material liquid contains a water-based gel used for hydration; thus, it is a powder/liquid material. There are few tricalcium silicate-based powder/liquid sealers, and this new formulation aims precisely to address this problem regarding the material hydration detected in ready-to-use sealers ^12^. There are no previous studies investigating this material.

Therefore, the present study aimed to evaluate the physical and chemical properties of a novel prototype material - MTApex Sealer in powder/liquid form - by comparing this material both to a premixed ready-to-use sealer and to an epoxy resin-based paste/paste sealer, especially regarding possible differences in the setting time due to their different material characteristics. The tested null hypothesis is that there are no differences in the tested sealers properties.

## Material and methods

### Preparation of the materials

Chemical composition and batch number of the tested materials are shown in Box 1.

**Box 1:**
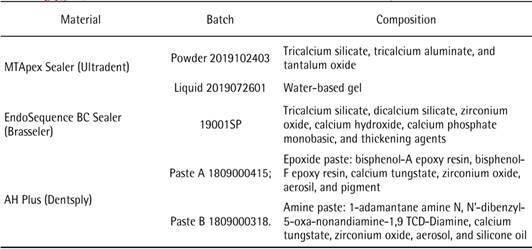
Composition and batch number of the root canal sealers used in the analysis.

MTApex Sealer mixing used a full spoon (provided by the manufacturer) of powder to 4 drops of the water-based gel for 60 seconds. EndoSequence BC Sealer is available in pre-mixed and ready-to-use form, without the need for mixing. AH Plus was mixed for 60 seconds by using pastes A and B at a 1:1 ratio, according to the manufacturer’s instructions and used as control. During analysis, all samples were kept in an oven at 37°C and 95% humidity, according to the ISO standard test requirements. The sample size was determined for each test to ensure a test power of at least 80% using the software Gpower 3.0 resulting in the sample amount used (n = 6).

### Setting time

Setting time was determined by using two different methods. The moist method used previously manufactured round plaster molds (Durone-IV; Dentsply, Rio de Janeiro, RJ, Brazil) (10 x 1 mm, according to ISO 6876-2012) ﬁlled with freshly mixed or pre-mixed sealers (n = 6). Every 30 minutes at first, and shorter than that in the final moments for precise determination, a 113.4 g Gilmore needle with a 2-mm tip was placed vertically onto the sample surface to determine the initial setting time. Then, a 453.6 g Gilmore needle with a 1.06-mm diameter tip (ASTM-C266-07) was used to determine the ﬁnal setting time ^9^. When the second-needle mark could no longer be observed on the material’s surface, the ﬁnal setting time was recorded. The dry method used stainless steel rings placed on a glass plate, and the tested sealers (n = 6) were inserted into the rings (10 x 2 mm). Next, a cotton moistened with distilled water was placed around the glass plate without contact with the sealers to provide the additional moisture for this analysis. The determination of setting time (in minutes) for the dry analysis samples continued using identical Gilmore needles, as previously described.

### Flow

For the flow analysis according to the ISO 6876:2012 standard, a chronometer was started, and the non-premixed materials were freshly prepared for the test. A volume of 0.05 mL of each sealer (n = 6) was placed in the center of a flat glass plate by using a graduated syringe (BD-Luer-Lok, MG, Brazil). Next, a second 20-g plate along with an additional 100-g weight was centrally placed on top of each sample. After 10 minutes from the beginning of the mixing, the additional weight was removed and the minimum and maximum diameters of each sample were measured by using a digital caliper (500-463; Mitutoyo Corporation, Kanagawa, Japan). The flow was determined, in millimeters, as the average value between the two diameters.

### Radiopacity

For radiopacity analysis, round samples (10 x 1 mm) of the sealers were prepared also according to ISO 6876:2012 standard. After their final setting time a 600-grit sandpaper was gently used for thickness standardization. The samples were radiographed (60 kV, 10 mA, 0.3 seconds, and focus-film distance of 30 cm) with a digital sensor (Micro-Image, Sao Paulo, Brazil) along with a 2 to 16 mm aluminum scale. Radiopacity was assessed by analyzing the grey levels (from 0 to 256) of the images obtained by using the Image Tool software (Version 3.0, University of Texas, Texas, USA), in which an area of 3,000 pixels was compared to the aluminum scale. The radiopacity values were expressed in millimeters of aluminum.

### pH analysis

The pH of the sealers was assessed after 1, 7, 14 and 21 days of immersion. The sealers were sampled by using polyethylene tubes (10 x 1.6 mm) and then immersed in plastic containers with 10 mL of deionized water, stored at 37°C and relative humidity of 95%. Next, pH was assessed in each period by using a previously calibrated digital pH-meter (Digimed, Sao Paulo, Brazil). The average pH of each sealer was obtained.

### Chemical surface characterization

After setting time, round samples (10-mm diameter x 1-mm thick) of each sealer were separately carbon coated for surface characterization by using scanning electron microscopy (SEM) (JSM 5600, JEOL, Japan) operating in backscatter mode. To determine the chemical composition of the sealers, SEM coupled with energy dispersive spectroscopy (EDS) was used. Representative SEM micrographs were obtained at 500x magnification and EDS analysis provided the chemical profile peaks for each material.

### Statistical analysis

JASP (University of Amsterdam, Amsterdam, The Netherlands), version 0.9.2. ^2020^ with the use of Mixed Anova (within and between the subjects' effects) software was used. Method's assumptions, normal distribution and sphericity of variances were validated through Shapiro-Wilk’s and Levene’s tests. A *post hoc* analysis with Bonferroni correction was also performed for differences between the groups. All statistical tests were performed at a significance level of 5% (α = 0.05). 

## Results

Results regarding setting time, flow and radiopacity are shown in Table 1.

**Table 1:** Representation of the mean and standard deviation values of setting time, flow and radiopacity of the tested materials.

Tested material	Setting time (min)		Flow	Radiopacity
	Moist method	Dry method	(mm)	mm Al
MTApex Sealer	1160.00 ± 20.49^Aa^	1382.50 ± 11.29^Aa^	30.43 ± 2.26^a^	7.99 ± 0.19^a^
EndoSequence BC sealer	490.00 ± 40.98^Ab^	4228.33 ± 76.20^Bb^	21.59 ± 4.10^b^	7.50 ± 0.17^b^
AH Plus	442.50 ± 18.37^Ac^	485.00 ± 27.92^Ac^	23.50 ± 1.35^b^	8.16 ± 0.36^a^

Different lowercase letters indicate statistically significant differences (p < 0.05). As for the setting time, uppercase letters indicate the differences between the methods for each sealer.

### Setting time

EndoSequence BC Sealer obtained a significantly longer setting time in the dry method when compared to the moist method (p = 0.0004). Regardless of the moist or dry method used, similar setting times results were observed for MTApex Sealer (p = 0.7913) and AH Plus (p = 0.7494). AH Plus had the fastest setting time for both methods, whereas EndoSequence BC Sealer exhibited the longest setting time in the dry method, and MTApex Sealer the longest setting time in the moist method.

### Flow

MTApex Sealer had the highest flow considering its hydration is performed using a water-based gel compared to the premixed EndoSequence BC Sealer (p = 0.0009) and to the paste/paste sealer AH Plus (p < 0.0001).

### Radiopacity

MTApex Sealer had a similar radiopacity compared to that of AH Plus (p = 0.3345) and a significantly higher than that observed for EndoSequence BC Sealer (p = 0.0009).

### pH analysis

The materials pH analysis for the 1 to the 21-day is shown in Table 2. All sealers had a significant decreasing in their pH values after 21 days in comparison to those on day 1. MTApex Sealer and EndoSequence BC Sealer exhibited significantly higher pH values in comparison to the AH Plus for all analysis periods (p < 0.001).

**Table 2:** Representation of the mean and standard deviation values of the pH analysis after 1, 7, 14, and 21 days of immersion.

Tested material	pH			
	1 day	7 days	14 days	21 days
MTApex Sealer	8.10 ± 0.04^Aa^	7.99 ± 0.10^Ba^	7.90 ± 0.05^Ba^	7.78 ± 0.03^Ca^
EndoSequence BC sealer	8.32 ± 0.10^Aa^	8.14 ± 0.08^Ba^	7.97 ± 0.05^Ca^	7.78 ± 0.09^Da^
AH Plus	7.62 ± 0.34^Ab^	7.37 ± 0.12^Bb^	7.27 ± 0.05^Bb^	7.21 ± 0.06^Bb^

Different letters indicate statistically significant differences between experimental periods (uppercase) and materials (lowercase) (p < 0.05).

### Chemical surface characterization

**Figure 1:**
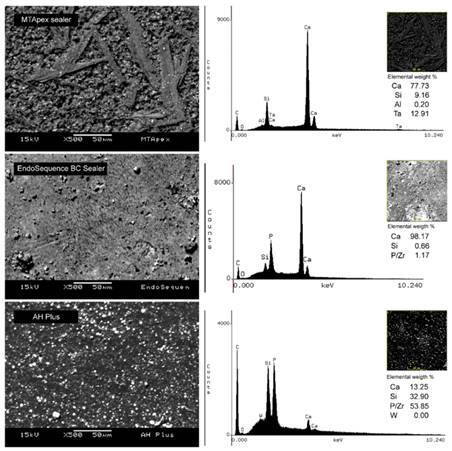
Representative SEM micrographs and EDS peaks with elemental weight of the tested sealers. MTApex Sealer showed stretched crystalline structures interposed within the matrix and peaks of calcium, silicon, aluminum, and tantalum. SEM micrographs of EndoSequence BC Sealer showing regular matrix with interposition of small particles. Under EDS, peaks of calcium and silicon were observed for this material, but zirconium and phosphorus overlapped. AH Plus showed a uniform matrix with interposition of different-sized particles. Under coupled EDS, a different peak pattern was observed in comparison to other materials, with peaks of calcium, silicon and tungsten being detected as well as zirconium and phosphorus overlap.

SEM micrographs and EDS chemical characterization of the tested sealers surfaces are shown in Figure 1. Under SEM analysis, MTApex Sealer exhibited stretched crystalline structures interposed within its matrix, whereas EDS analysis showed calcium, silicon, aluminum, and tantalum peaks, with calcium ions representing the highest atomic weight percentage. For EndoSequence BC Sealer, a uniform matrix with interposition of small particles was observed. Although peaks of calcium and silicon were observed, those of zirconium and phosphorus overlapped, which is a limitation of the EDS technique when these two ions are mapped. For this material, calcium also represented the highest atomic weight percentage. Finally, AH Plus showed a uniform matrix with interposition of different-sized particles. The EDS analysis obtained a different peak pattern when compared to that of other materials. Peaks of calcium, silicon and tungsten were detected; also, zirconium and phosphorus overlapped for this material, with the highest atomic weight percentage being represented for this ion overlap.

## Discussion

In the present study, significant differences between the tested calcium silicate-based sealers in powder/liquid and ready-to-use forms were observed, which lead to a rejection of the experimental hypothesis of similarity between these materials presentations. The powder/liquid form exhibited significantly more predictable results, especially regarding its setting time. *In vitro* analysis of root canal endodontic sealers properties is crucial for predicting the clinical behavior of newly-develop materials. Although, a limitation of this study can be the fact that a humidity artificially provided may not represent the biological scenario, but it gives a general idea of the behavior of these materials prior to its clinical use, especially considering its setting.

The setting time of an endodontic sealer ideally must allow proper handling time for obturation to warrant a good quality root canal filling and, also, set quickly to ensure the material’s long-term properties ^13^. It is known that when a premixed sealer based on calcium silicate is used, its hydration reaction does not start until the material contacts either the residual moisture inside the root canal or the dentinal tubules humidity ^14^. Therefore, it is essential to evaluate the setting time *in vitro* under the ISO 6876-2012 standard, which recommends testing in moist and dry conditions considering that hydration supposedly occurs inside the root canal in the presence of moisture. In the present study, MTApex Sealer - in which hydration reaction initiate prior to its insertion into the root canal - no difference was observed between dry and moist methods regarding its setting time. This is clinically relevant, as it is questioned if moisture from dentinal tubules is enough to initiate and complete the setting of ready-to-use calcium silicate-based sealers ^7^. The results clearly demonstrate that powder-to-liquid formulation has an advantage in guaranteeing the setting and, consequently, the adequate performance of the sealer. EndoSequence BC Sealer showed a significantly longer setting time (approximately 10-fold) when the dry method was considered in comparison to the moist method. Even under *in vitro* controlled moist, the premixed sealer obtained a long setting time, which corroborates with this concern previously raised in the literature ^14^. This fact should be highly regarded in the clinical use of premixed sealers; especially when drying the root canal before filling, since the amount of residual moisture may crucially and critically interfere with the sealer’s properties. Moreover, a non-set material would not be able to exert its sealing properties, and consequently, a clinical failure would be expected ^6^. In contrary, considering the AH Plus, no difference was observed in setting time between the methods, considering that epoxy resin-based sealers set independently of moisture.

The flow properties of endodontic sealers can be considered critical during obturation procedures as sealers must have abilities to achieve and seal the complex anatomical structures of the root canal ^3^. MTApex Sealer showed the highest flow compared to the other tested materials, probably due to the water-based gel used for its hydration. However, all the materials obtained values above 17 mm, which meets the recommendations of ISO 6876/2012 for endodontic sealers. These findings corroborate with previous studies ^15^ that reported similar flow values for EndoSequence BC Sealer and AH Plus. To date, no other study has evaluated the MTApex Sealer regarding this property.

Radiopacity is a property in which endodontic filling materials can be viewed on radiographic and tomographic images after clinical insertion and thus allowing to verify the filling quality and its related long-term outcomes ^8^. Therefore, radiopacity is a valuable property for evaluation of the new sealers' compositions. In the present study, all the investigated materials showed radiopacity above 7 mm Al, thus meeting the ISO 6876/2012 standard. MTApex Sealer and AH Plus obtained significantly higher values than EndoSequence BC Sealer. These radiopacity values of MTApex Sealer may be related to the tantalum oxide present in the powder of this material as radiopacifier. AH Plus and EndoSequence BC Sealer compositions use zirconium oxide as a radiopacifier in their compositions, although their manufacturers do not declare the exact amount and proportion of these components. Moreover, AH Plus sealer contains not only zirconium oxide, but also calcium tungstate as a second radiopacifier, which probably justify the significantly higher results of radiopacity in comparison to the EndoSequence BC Sealer.

The long-term dimensional stability of endodontic sealers is essential to ensure apical sealing and prevent microorganisms from invading the root canals, thus resulting in successful endodontic treatment ^16^. Although the solubility of calcium silicate-based sealers may be considered a disadvantage, its biological reparative potential is a consequence of this solubility after setting. The alkaline pH observed after hydration is due to calcium and hydroxyl ions released from the sealer’s matrix, which are potentially associated with its biological properties ^17^. All tested sealers showed a decreasing alkaline pH towards the 21-day analysis. AH Plus sealer was previously reported ^18^ to have an acidic pH; however, our results obtained a pH above 7 for this sealer at all times. MTApex Sealer had results comparable with those found in a previous study ^19^ which used another endodontic sealer (BioRoot RCS, Septodont) in a similar powder/liquid form once they have a similar main chemical component (i.e. tricalcium silicate). Therefore, it is expected that these materials obtain a higher pH in solution due to hydroxyl and calcium ion release ^20^. No previous studies evaluated MTApex Sealer regarding its pH.

Surface characterization and ionic identification of the sealers' composition after its hydration or mixing are crucial to evaluate uniformity and distribution of the particles. SEM coupled with EDS is a well-established method for evaluating hydraulic calcium silicate-based sealers ^21^. In the present study, the calcium silicate-based sealers showed peaks of calcium, silicon and different radiopacifiers. MTApex Sealer had peaks of tantalum, which is a component added to the material’s composition as a radiopacifier. Traces of aluminum were also detected in the MTApex Sealer samples, which corroborates a previous study ^22^ investigating calcium silicate-based endodontic materials and described the presence of aluminum in these compositions. EndoSequence BC Sealer and AH Plus exhibited calcium, silicon, and zirconium used as radiopacifiers, as stated by their manufacturers. Additionally, AH Plus showed a uniform matrix and particles, which can be attributed to its epoxy resin-based composition. However, peaks of zirconium and phosphorus overlapped in both materials, a fact that was previously described ^23^ as a limitation of EDS analysis for these two ions for hydraulic endodontic materials, once it is impossible to separate their percentage atomic weights using this analysis. Further studies should use different methods to identify compounds when zirconium is present in the material’s composition.

MTApex Sealer showed the highest flow without changes in the setting time regardless of the additional moisture, suggesting that the powder/liquid form increased the materials’ setting predictability. Considering the clinical scenario, the endodontic sealers immediate behavior towards setting and its long-term stability is highly expected to ensure their successful clinical outcomes.

## References

[1] Schilder H (2006). Filling Root Canals in Three Dimensions. J Endod.

[2] Komabayashi T, Colmenar D, Cvach N, Bhat A, Primus C, Imai Y (2020). Comprehensive review of current endodontic sealers. Dent Mater J.

[3] Antunes TBM, Janini ACP, Pelepenko LE (2021). Heating stability, physical and chemical analysis of calcium silicate‐based endodontic sealers. Int Endod J.

[4] Duarte MAH, Marciano MA, Vivan RR, Tanomaru M, Tanomaru JMG, Camilleri J (2018). Tricalcium silicate-based cements: properties and modifications. Braz Oral Res.

[5] Loushine BA, Bryan TE, Looney SW (2011). Setting Properties and Cytotoxicity Evaluation of a Premixed Bioceramic Root Canal Sealer. J Endod.

[6] Camilleri J (2020). Classification of Hydraulic Cements Used in Dentistry. Front Dent Med..

[7] Zmener O, Spielberg C, Lamberghini F, Rucci M (1997). Sealing properties of a new epoxy resin-based root-canal sealer. Int Endod J.

[8] Zavattini A, Knight A, Foschi F, Mannocci F (2020). Outcome of Root Canal Treatments Using a New Calcium Silicate Root Canal Sealer: A Non-Randomized Clinical Trial. J Clin Med.

[9] Chen B, Haapasalo M, Mobuchon C, Li X, Ma J, Shen Y (2020). Cytotoxicity and the Effect of Temperature on Physical Properties and Chemical Composition of a New Calcium Silicate-based Root Canal Sealer. J Endod.

[10] Rodríguez‐Lozano F, López‐García S, García‐Bernal D (2020). Chemical composition and bioactivity potential of the new Endosequence BC Sealer formulation HiFlow. Int Endod J. May.

[11] Pelepenko LE, Saavedra F, Antunes TBM (2021). Physicochemical, antimicrobial, and biological properties of White-MTAFlow. Clin Oral Investig.

[12] Xuereb M, Vella P, Damidot D, Sammut C V., Camilleri J (2015). In Situ Assessment of the Setting of Tricalcium Silicate-based Sealers Using a Dentin Pressure Model. J Endod.

[13] Li G, Niu L, Zhang W (2014). Ability of new obturation materials to improve the seal of the root canal system: A review. Acta Biomater.

[14] Ozlek E, Gündüz H, Akkol E, Neelakantan P (2020). Dentin moisture conditions strongly influence its interactions with bioactive root canal sealers. Restor Dent Endod.

[15] Candeiro GT de M, Correia FC, Duarte MAH, Ribeiro-Siqueira DC, Gavini G (2012). Evaluation of Radiopacity, pH, Release of Calcium Ions, and Flow of a Bioceramic Root Canal Sealer. J Endod..

[16] Ørstavik D, Nordahl I, Tibballs JE. (2001). Dimensional change following setting of root canal sealer materials. Dent Mater.

[17] Donnermeyer D, Bürklein S, Dammaschke T, Schäfer E (2019). Endodontic sealers based on calcium silicates: a systematic review. Odontology.

[18] Zordan-Bronzel CL, Esteves Torres FF, Tanomaru-Filho M, Chávez-Andrade GM, Bosso-Martelo R, Guerreiro-Tanomaru JM (2019). Evaluation of Physicochemical Properties of a New Calcium Silicate-based Sealer, Bio-C Sealer. J Endod.

[19] Urban K, Neuhaus J, Donnermeyer D, Schäfer E, Dammaschke T (2018). Solubility and pH Value of 3 Different Root Canal Sealers: A Long-term Investigation. J Endod.

[20] Koutroulis A, Kuehne SA, Cooper PR, Camilleri J (2019). The role of calcium ion release on biocompatibility and antimicrobial properties of hydraulic cements. Sci Rep.

[21] Marciano MA, Duarte MAH, Camilleri J (2016). Calcium silicate-based sealers: Assessment of physicochemical properties, porosity and hydration. Dent Mater.

[22] Viapiana R, Guerreiro-Tanomaru JM, Hungaro-Duarte MA, Tanomaru-Filho M, Camilleri J (2014). Chemical characterization and bioactivity of epoxy resin and Portland cement-based sealers with niobium and zirconium oxide radiopacifiers. Dent Mater.

[23] Moinzadeh AT, Aznar Portoles C, Schembri Wismayer P, Camilleri J (2016). Bioactivity potential of endo sequence BC RRM putty. J Endod.

